# Noninvasive Metabolomic Profiling of Human Embryo Culture Media Using a Simple Spectroscopy Adjunct to Morphology for Embryo Assessment in *in Vitro* Fertilization (IVF)

**DOI:** 10.3390/ijms14046556

**Published:** 2013-03-25

**Authors:** Qinghong Zhao, Tailang Yin, Jin Peng, Yujie Zou, Jing Yang, Aiguo Shen, Jiming Hu

**Affiliations:** 1 Reproductive Medical Center, Renmin Hospital of Wuhan University, JieFang Road 238, Wuhan 430060, China; E-Mails: zhaoqinghong_0815@126.com (Q.Z.); reproductive@126.com (T.Y.); yujiezou11@163.com (Y.Z.); 2 College of Chemistry and Molecular Science, Wuhan University, Wuhan 430072, China; E-Mails: pengjin_zhao@163.com (J.P.); agshen@whu.edu.cn (A.S.)

**Keywords:** metabolomics, Raman spectroscopy, culture media, IVF

## Abstract

Embryo quality is crucial to the outcome of *in vitro* fertilization (IVF); however, the ability to precisely distinguish the embryos with higher reproductive potential from others is poor. Morphologic evaluation used to play an important role in assessing embryo quality, but it is somewhat subjective. The culture medium is the immediate environment of the embryos *in vitro*, and a change of the substances in the culture medium is possibly related to the embryo quality. Thus, the present study aims to determine whether metabolomic profiling of the culture medium using Raman spectroscopy adjunct to morphology correlates with the reproductive potential of embryos in IVF and, thus, to look for a new method of assessing embryo quality. Fifty seven spent media samples were detected by Raman spectroscopy. Combined with embryo morphology scores, we found that embryos in culture media with less than 0.012 of sodium pyruvate and more than −0.00085 phenylalanine have a high reproductive potential, with up to 85.7% accuracy compared with clinical pregnancy. So, sodium pyruvate and phenylalanine in culture medium play an important role in the development of the embryo. Raman spectroscopy is an important tool that provides a new and accurate assessment of higher quality embryos.

## 1. Introduction

Since the first successful pregnancy using *in vitro* fertilization (IVF) was reported in 1978, human assisted reproductive technology (ART) has made considerable progress. Infertility was estimated to affect 15% of the reproductive aged population [1]. Although ART has brought hope to these couples, the multiple pregnancies that resulted from ART caused several serious problems and also constituted a health risk for the mother and fetus. The main risks for the mother include abortion, pregnancy-induced hypertension and postpartum hemorrhage [2], whereas those for the fetus include preterm birth and low birth weight [3]. Moreover, multiple pregnancies could yield a huge medical and social burden [4].

Why does multiple pregnancy occur so frequently? In natural pregnancy, the twin pregnancy rate is only 1/80 and that of three pregnancies is 1/6400. However, in the ART cycle, the multiple pregnancy rate is significantly higher, reaching 20%–40% or even 50% [5]. This phenomenon is largely due to our inability to predict the potential of an embryo. Morphological evaluation is the currently used embryo grading system, which is largely based on embryo cleavage rate and morphology [6,7]. However, this system is extremely subjective and insufficiently precise in clinical diagnosis. As we are unable to predict embryo potential, IVF centers have historically chosen to transfer multiple embryos simultaneously to avoid implantation failure and achieve a relatively high pregnancy rate. This approach led to two major problems in IVF: a low implantation rate and high multiple pregnancy rate. Consequently, decreasing multiple gestations, as well as maintaining or increasing overall pregnancy rates, is a significant contemporary goal of infertility treatment [8].

Embryo quality is an important factor affecting the clinical outcome of ART. As the culture medium is the direct environment of the embryo *in vitro*, it would be a new approach to predict the potential of the embryo by detecting changes in the composition of the culture medium. Recently, several scientists have studied the culture environment and the metabolic characteristics of the embryo itself. For example, Brison [9] measured the amino acid turnover to assess embryos. However, these methods require specialized technical personnel and are time-consuming, so they have not been applied in clinical practice.

Raman spectrometry, measuring the vibrations of bonds, is an optical spectroscopy method that has gradually drawn scientists’ interest for profiling embryo culture media in relation to pregnancy outcome. There are some advantages, including sample processing, high throughput and rapid turnaround. The present study was intended to assess the metabolomic profile of embryo culture media using Raman spectroscopy and to examine the capability of predicting pregnancy outcome using this technique compared with morphologic methods. We aimed at determining a rapid, noninvasive method for the assessment of the developmental potential of embryos.

## 2. Results and Discussion

**Figure 1:**
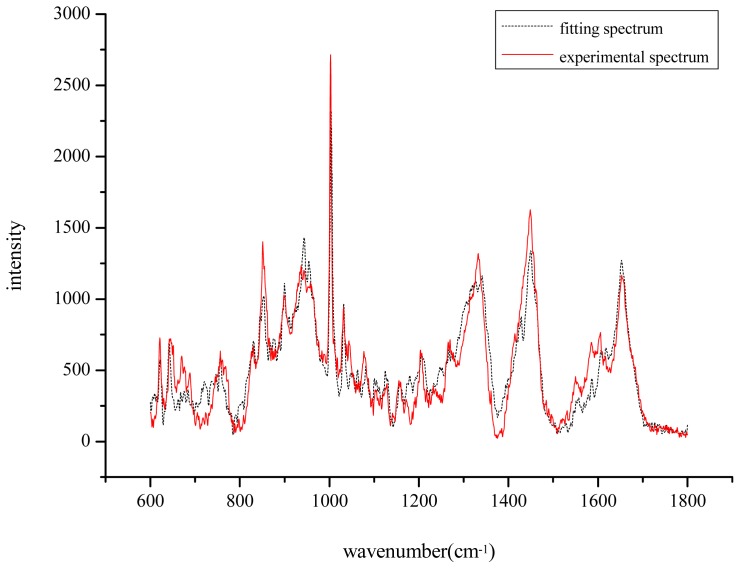
The average spectrum of the samples( 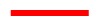) and the fitting spectrum(…).

Raman spectra were obtained from the following commercially available chemicals (Wuhan Boster Biological Technology Ltd., Wuhan, China) for use in spectral fitting and Raman criterion development: albumin, monopotassium phosphate, cysteine, histidine, phenylalanine, methionine, serine, tyrosine, tryptophan sodium, pyruvate, sodium citrate and taurine. The fitting spectra was calculated using a self-produced program in the Matlab software platform (MathWorks, Natick, MA, USA). Figure 1 shows that the fitting spectra shared great similarity with the measured Raman spectra of the samples. The average spectrum of each sample was calculated using the approach above, obtaining the fitting coefficient of the corresponding components involved in the medium as the relative concentration (C′).

It can be seen from Figure 1 that some degree of faults for similarities still exist, due to the calculation error using the least squares method in Matlab during the fitting spectrum. Actually, the self-built program can only minimize the error as much as possible during the process of calculation.

### 2.1. A Preliminary Analysis of the 45 Samples

Based on the theory above, the Raman spectra of 45 samples from six patients were detected and analyzed with the Matlab software. In comparison, we found that the relative concentrations (C′) of sodium pyruvate and phenylalanine showed a certain degree of regularity.

**Figure 2:**
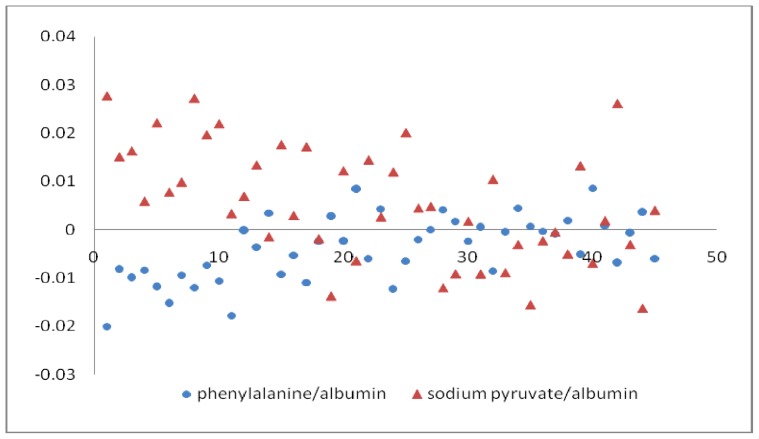
The distribution of 45 samples according to the two relative concentrations.

The currently used embryo grading systems were developed soon after the report of the first successful pregnancy after IVF [10]. Although their accuracy remained insufficient, these grading systems led to significant improvements in the implantation rate and pregnancy rate [11], such that morphological assessment is the first-line approach for embryo selection. The establishment of Raman criterion to screen the higher quality of embryos should obey the results of morphological assessment. Based on the morphological assessment, we obtain the threshold of pyruvate/albumin and phenylalanine/albumin to be lower than 0.012 and higher than −0.00085, respectively. The results are shown in Figure 2 and Table 1.

**Figure 3:**
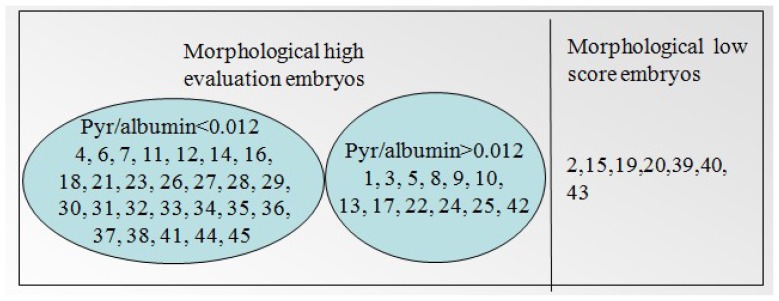
The result of 45 samples according to the morphology scoring and pyruvate/albumin relative fitting coefficients.

**Figure 4:**
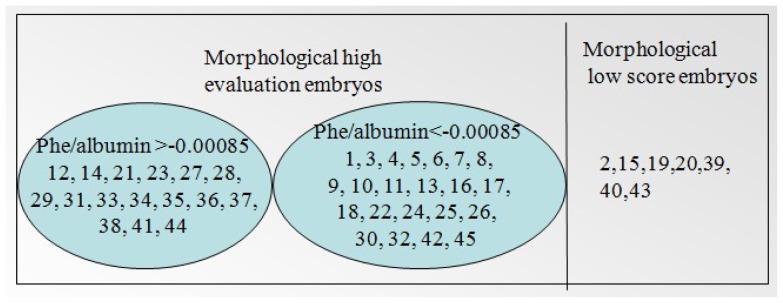
The result of 45 samples according to the morphology scoring and phenylalanine/albumin relative fitting coefficients.

By analyzing the Raman information of the samples, combined with the embryo morphology scores, we found that the threshold for the relative concentration of sodium pyruvate was 0.012; that is, the embryos in culture media with less than 0.012 had high reproductive potential, whereas those with more than 0.012 had low developmental potential. For phenylalanine, the threshold is −0.00085; that is, embryos in media with phenylalanine levels above −0.00085 had high developmental capacity, whereas those in media with levels below −0.00085 had low developmental capacity. These results are expressed in detail in Figures 3 and 4.

**Figure 5:**
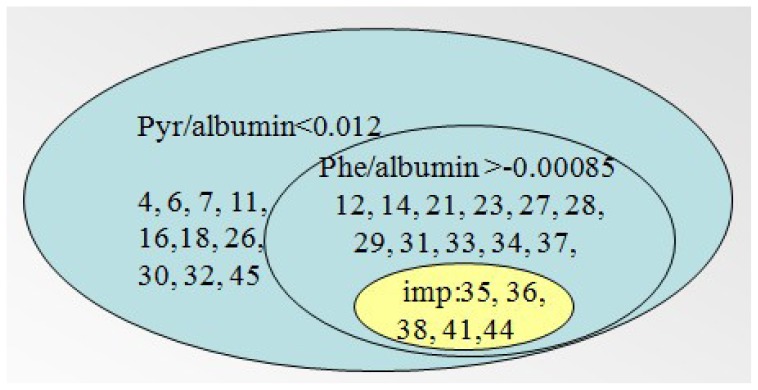
The result of 45 samples using pyruvate/albumin and phenylalanine/albumin relative fitting coefficients (imp = implanted).

A total of 16 labels met the two conditions above simultaneously and were considered to have high developmental potential: 12, 14, 21, 23, 27, 28, 29, 31, 33, 34, 35, 36, 37, 38, 41 and 44. The labels of the corresponding embryos transferred were 12, 21, 23, 27, 29, 35, 36, 38, 41 and 44; the remainder of the embryos were cryopreserved, with the patients’ agreement. The labels of the embryos that resulted in clinical pregnancy were 35, 36, 38, 41 and 44, all of which were in the range of the embryos with high developmental potential by the previous test (Figure 5).

### 2.2. The Verification of Preliminary Results

Of the second batch of 12 samples from four patients numbered 46–57, after removing the lower scoring embryos based on morphological evaluation, the remaining samples were detected by Raman spectroscopy in accordance with the methods above and the metabolism of the spent culture media was analyzed. We found that embryos with a better ability to develop corresponded to the labels 46, 47, 49, 50, 52, 53 and 56. The labels of the embryos that achieved pregnancy in four patients were 46, 47, 49, 50, 52 and 53. Thus, Raman spectroscopy combined with morphological methods to assess early embryo quality could achieve an accuracy rate up to 85.7% (6/7).

In this study, we analyzed the spent culture media of embryos with known outcomes after embryo transfer (ET) on day three. First, we assessed the embryos using the current embryo selection methodology based on cleavage rate and morphology; then, we excluded the spent culture media of the embryos with lower scores. The remaining samples were detected using Raman spectroscopy under certain conditions, and high-quality embryos were selected based on the relative concentrations of pyruvate sodium and phenylalanine. Our results suggest that the metabolomic profiles of embryonic development in IVF may associate with the ability to implant. Additionally, compared with embryos that failed in a pregnancy, *in vitro* cultured embryos with a high reproductive potential displayed a difference in the alteration of the culture environment that was detectable using spectroscopy.

During early cleavage, pyruvate is the preferred energy substrate for embryos [12], whereas glucose becomes the primary energy source at the blastocyst stage [13]. We also observed a trend toward lower pyruvate levels in the day three culture media of embryos that resulted in pregnancy compared with those that failed to implant (Table 1). As the energy in the cleavage stage is provided mainly by the citric acid cycle, pyruvate consumption in embryo culture media can be regarded as an indicator of embryonic vitality and developmental potential. Our findings are consistent with previous reports by Hardy [14] and Conaghan [15].

There are large quantities of free amino acids in oviductal and uterine fluid [16,17], which are important regulatory factors in the process of embryo culture [18], including acting as prerequisites for the biosynthesis of cells, being involved in carbohydrate metabolism and regulating the cell osmotic pressure and pH. Amino acids can maintain the normal function of cells and improve the implantation. Gardner proved that the ideal environment for embryonic development included the presence of amino acids. Early embryonic development requires several specific amino acids, and after embryonic genome activation, all 20 amino acids are needed. Based on these results, Gardner and coworkers [19] produced two sequential media, called G1 and G2. Non-essential amino acids and essential amino acids play different roles in the different developmental stages of embryos. Non-essential amino acids can promote the embryonic development from the cleavage stage to the blastocyst, increase the number of trophoblast cells and enhance the ability of blastocyst hatching. Essential amino acids, however, mainly increase the division rate of the inner cell mass and improve the ability of fetal development after implantation. Houghton [20] and his colleagues hold that amino acid turnover can predict human embryo developmental capacity using high-performance liquid chromatography (HPLC). Brison [9] used the same approach and found that the combination of decreased glycine and leucine and increased asparagine levels in the culture medium correlates with increased clinical pregnancy rate, and Sturmey [21] reported similar findings in cryopreserved embryos. Seli [22] found an association between higher glutamate levels in the culture medium and clinical pregnancy rates using proton nuclear magnetic resonance (^1^H NMR). In the present study, we found that phenylalanine levels in the spent culture media samples of embryos that resulted in pregnancy were higher than those of embryos that fail to implant (Table 1).

The development of embryo grading systems based on cleavage rate and morphology [23–26] led to significant improvements in implantation and pregnancy rate and reductions in multiple gestation rate [11]. The morphological evaluation method is rapid and non-invasive, but still subjective, and its precision is insufficient to enable most patients and clinicians to reduce the number of embryos transferred. These limitations have led many investigators to pursue adjunctive technologies for determining an individual embryo’s reproductive potential.

Other methods for the improvement of IVF outcomes are currently being studied. Preimplantation genetic screening has been proposed as a technique to improve embryo selection in specific populations, such as patients of older age or with repeated miscarriages. However, the effectiveness of this technique is still questionable [27]. In addition, routine preimplantation genetic screening is time-consuming, requires considerable resources and is not suitable for all patients. Although there are many other methods to analyze the metabolism of embryos, there are limitations in their clinical application to various extents. For example, many of these technologies require complex equipment and dedicated technical staff that are not available at most embryology laboratories, and several technologies are so time-consuming as to miss the limited window of time available for embryo transfer.

The commercial culture media used in ART laboratories all share the same components, as they are all based on the metabolic requirements of human embryos [28]. We chose Single Step Medium™ (SSM) culture medium in our study. The culture medium is the direct environment for embryo development *in vitro*, which is very important for the physiology and viability of the pre-implanted embryos [29]. Thus, the analysis of embryo culture medium can predict developmental potential noninvasively and objectively, and this draws public attention. The current detection methods include HPLC [20], near-infrared (NIR) [30] and ^1^H NMR [22]. Ahlstrom [31] in Reproductive Biomedicine 2011 demonstrated that metabolomics profiling by NIR spectroscopy analysis can predict the implantation potential of blastocysts. Nadal-Desbarats [32] and coworkers in MAGMA 2012 used ^1^H NMR metabolomics profiling on 15 micro-L of embryo culture medium with multivariate data analysis to predict embryo viability. However, these technologies require complex equipment and preparation of samples, professional inspection and a long testing time that exceeds the time limit for embryo selection, which have been the limiting factors in routine viability assessment. As a result, the search for an accurate, rapid and noninvasive assessment is becoming an urgent problem to be solved in reproductive medicine.

Most recently, it was discovered that the oxidative status of the early embryo in IVF, as assessed by a thermo-chemiluminescence (TCL) analyzer, was associated with the chances of implantation; this approach was also applicable for centers that routinely perform elective transfer of two embryos [33]. The ultimate aim is to find a suitable technology to assess embryo quality quickly for clinical practice.

Raman spectroscopy is based on molecular vibration information and can provide a wealth of molecular structure and composition information without causing any damage. Moreover, Raman spectroscopy is especially applicable for aqueous samples, because water only produces a very weak signal. Thus, this technique has become an important means of disease detection and diagnosis. In our study, it only took several minutes and 5 μL of sample volume to assess a sample using the chosen parameters. Raman is a simple, objective, real-time, noninvasive method.

Seli [34] and Scott [35] found that the viability index calculated by Raman spectroscopy was higher for embryos that succeed in implantation and delivery, compared with those that failed to implant. They analyzed individual samples using 15 μL of media, and spectra were recorded from 50 to 3450 cm^−1^. In contrast, our study sample size was as less as 5 μL. In addition, the spectral range was narrower, from 600 to 1800 cm^−1^. In particular, viability models used contributions from the wavelength regions of –SH, –CH and –NH, while our final analysis concluded that the specific substances in the culture medium, phenylalanine and pyruvate, are obviously related to the potential of embryos. Grading systems of embryo were developed by several investigators, leading to significant improvements in implantation and pregnancy rate, so they are always first-line approaches for embryo selection. The establishment of Raman criterion to screen the higher quality of embryos should be in accordance with the results of morphological assessment. In this study, to detect embryo culture medium using Raman spectroscopy combined with embryo morphology score, our method not only obviously reduces the scope in comparison with morphological assessment results, but it also accurately predicts clinical pregnancy.

## 3. Experimental Section

### 3.1. Patient Selection, Treatment and Sample Collection

#### 3.1.1. Patient Selection and Treatment

Institutional review board approval was obtained before the initiation of the study. All of the patients undergoing IVF signed an informed consent form. The patients who were more than 35 years old or who exhibited an endometrial thickness of less than 0.6 cm on the day of human chorionic gonadotropin (hCG) administration were excluded from the analysis.

The women underwent a long protocol treatment with gonadotropin-releasing hormone (GnRH)-agonist administration in the mid-luteal phase, which was followed by ovarian stimulation by recombinant follicle-stimulating hormone (FSH) (Gonal-f; Merck Serono, Geneva, Switzerland or Puregon; Schering-Plough; Kenilworth, NJ, USA) or hMG (human menopausal gonadotropin) (LIVZON Group, Guangzhou, China) when ovarian suppression was confirmed. Human chorionic gonadotropin (10,000 IU SC) (LIVZON Group, Guangzhou, China) was administered when at least three follicles reached a mean diameter of 18 mm under transvaginal ultrasound examination. Then, 34 to 36 h later, the oocytes were retrieved by ultrasound-guided transvaginal puncture. The insemination of the oocytes was performed with the use of standard IVF or intracytoplasmic sperm injection (ICSI) procedures, according to the appropriate indication, as previously described [36]. Fertilization was confirmed 16 to 20 h after oocyte retrieval by the presence of two distinct pronuclei under the inverted microscope. The zygotes were then placed individually into fresh 30 mL droplets of culture medium (Single Step Medium™ (SSM), Irvine Scientific, Dublin, Ireland), covered with mineral oil and maintained in an incubator at 37 °C under 5% CO_2_, 5% O_2_ and 90% N_2_ until embryo transfer. Embryo selection was performed according to the standard morphologic assessment methods published by Cummins [37], which are based on cleavage rate and morphology [7,8]. The embryo with the highest number of blastomeres and the least fragmentation was transferred 72 h after oocyte aspiration. The intramuscular luteal supplementation consisted of 80 mg/day micronized progesterone (P) (Utrogestan; Ferring) for 12 days. The number of embryos transferred in each cycle was determined based on the clinical assessment, in accordance with the recommendations of the Chinese Ministry of Health. Pregnancy was determined when the serum β-hCG level was >10 MIU/mL on day 12 after ET and clinical pregnancy with one or more gestational sacs was demonstrated under transvaginal ultrasound examination. The basic clinical and demographic characteristics of the patients were shown in Table 2.

#### 3.1.2. Sample Collection

After the removal of the embryos, the spent media were placed individually into labeled cryovials and then labeled again with a randomly assigned accession number. The collected specimens were immediately frozen and stored at −20 °C. A total of 57 spent embryo culture medium samples were collected. A control sample (CM) incubated under the same conditions without an embryo was also collected and used for normalization. The morphological classification of the embryos in this experiment was described in detail in Table 3. The embryos with cell count ≥6 and class A or B are considered as morphologically high-scored embryos, otherwise, the embryos with cell count <6 and class A or B or cell count ≥6, but grade C, are considered as morphologically low-scored embryos.

SM is a commercial embryo culture medium that contains the necessary nutrition for embryonic development, such as inorganic salts, energy materials and essential amino acids. The specific composition and concentration of SSM are shown in Table 4. The concentrations of all components were located in the range of the mM level. Raman spectra were obtained in the 600–1800 cm^−o^ region.

### 3.2. Raman Analysis

**Figure 6:**
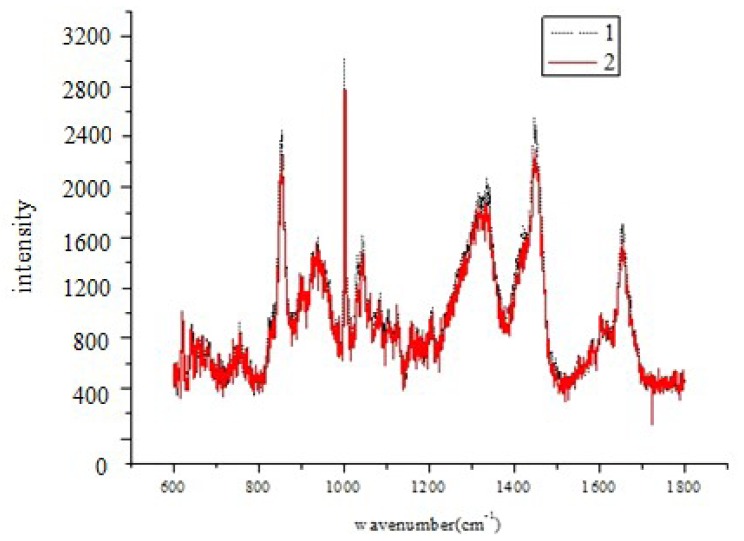
The average spectrum of spent culture media after the removal of the mineral oil layer using capillary siphoning (1) and the average spectrum of control samples (MC) (2).

Samples were thawed at room temperature (25 °C ± 1 °C) for 30 min. Before analyzing, the mineral oil on the surface of the spent media was removed by capillary siphon until there was no visual stratification. The mineral oil could prevent moisture from volatilizing during the process of embryo culture. The comparison of the spectra between the control samples (MC) and the sample after removing mineral oil are shown in Figure 6. The physical siphon turned out to be effective. Next, 5 μL of embryo culture medium, taken from the cryovials by pipette gun, were dropped into a round glass with a thin layer of gold film on the surface, and the glass was placed onto a slide.

Raman analysis was conducted using a HeNe-based Raman system. Spectra were recorded from 600 to 1800 cm^−1^. The signal acquisition time was 60 seconds, and the points used were sampled 5 times. Raman spectroscopy was collected at 10 to 15 points per sample.

Laser Raman spectroscopy of 57 samples from ten patients were conducted under the parameters above. The original spectra were exported in TXT form and then preprocessed automatically by the NGSLabSpec software built-in Raman instrument.

**Figure 7:**
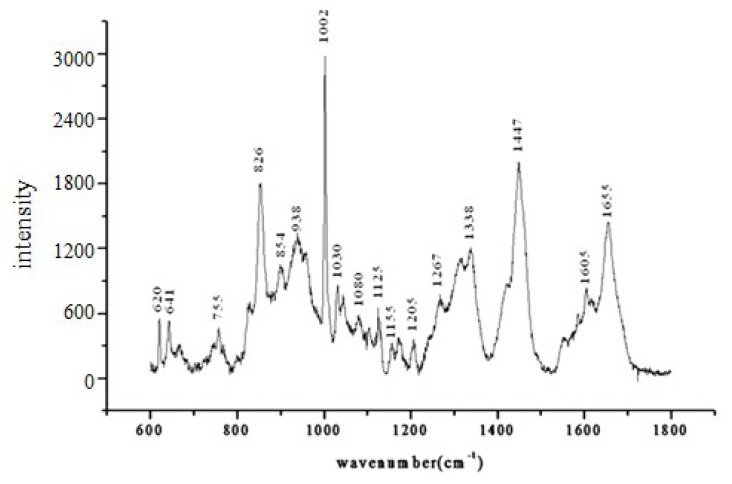
The average spectrum of all samples.

From the above figure, the Raman spectrum of the composition of the culture medium can be clearly observed in the range of 600–1800 cm^−1^. Raman spectrum peaks occur at 1002, 1030, 1447, 1655 cm^−1^, *etc*. (Figure 7). The peak at 1002 cm^−1^ should be assigned to the phenylalanine ring symmetrical vibration [38], which has high peak intensity, so that it has the highest sensitivity.

## 4. Conclusions

In this present study, we used Raman spectroscopy combined with morphology for the metabolomic profiling of human embryo culture media, and the overall accuracy for predicting a positive outcome was 85.7% (6/7), which suggested that the method may be an effective clinical tool for embryo selection. The number of transplanted embryos could be reduced when the accuracy of the assessment method was improved. Without any doubt, a good evaluation method can improve the embryo implantation rate and pregnancy rate and can lead to a decrease in the cost, as well as ultimately contributing to a decrease in multiple infants resulting from ART. To substantiate our results, further large-scale prospective randomized studies should be conducted comparing with current embryo selection methods based on spectroscopy alone.

## Figures and Tables

**Table 1:** The results according to the relative concentration (C′) of sodium pyruvate and phenylalanine.

Relative concentration (C′)	Range of C′	Embryo developmental capacity	Number of sample
Sodium pyruvate/albumin	<0.012	+	4, 6, 7, 11, 12, 14, 16, 18, 21, 23, 26, 27, 28, 29, 30, 31, 32, 33, 34, 35, 36, 37, 38, 41, 44, 45
	>0.012	—	1, 3, 5, 8, 9, 10, 13, 17, 22, 24, 25, 42

Phenylalanine/albumin	≥0.00085	+	12, 14, 21, 23, 27, 28, 29, 31, 33, 34, 35, 36, 37, 38, 41, 44
	≤0.00085	—	1, 3, 4, 5, 6, 7, 8, 9, 10, 11, 13, 16, 17, 18, 22, 24, 25, 26, 30, 32, 42, 45

**Table 2:** Clinical and *in vitro* fertilization (IVF) patients’ characteristics (Mean and SD).

Age (years)	Endometrial thickness (cm)	No. of oocytes retrieved	No. of high-quality embryos	Mean No. of transferred embryos per cycle
30.02 ± 3.12	0.93 ± 0.42	13.52 ± 4.10	9.73 ± 4.82	2.16 ± 0.78

**Table 3:** The morphological classification of embryos.

Sample number	The number of embryonic	Morphological classification
1	8	A
2	5	C
3	8	B
4	8	A
5	8	A
6	8	A
7	8	B
8	8	A
9	8	B
10	6	B
11	8	B
12	8	B
13	6	B
14	8	B
15	6	C
16	8	B
17	8	B
18	5	B
19	8	C
20	5	C
21	7	B
22	4	B
23	4	B
24	7	B
25	8	A
26	8	B
27	8	B
28	7	B
29	6	B
30	5	B
31	6	B
32	3	B
33	5	B
34	5	B
35	7	B
36	7	B
37	5	B
38	8	B
39	4	C
40	3	C
41	7	B
42	8	B
43	4	C
44	7	B
45	7	B
46	8	B
47	6	B
48	8	C
49	8	A
50	8	A
51	—	fragment
52	8	B
53	8	B
54	4	B
55	8	B
56	8	B
57	8	C

**Table 4:** The specific composition and concentration of Single Step Medium™ (SSM) embryo culture medium.

Component	Concentration (mM)
Sodium bicarbonate	25.0
Potassium chloride	2.5
Potassium phosphate	0.35
Calcium chloride, anhydrous	1.7
Sodium chloride	101.5
Magnesium sulfate, anhydrous	0.2
Sodium bicarbonate	25.0
Sodium pyruvate	0.2
Glucose	0.5
Sodium citrate	1.0
Sodium lactate (D/L)	20
EDTA, disodium, dihydrate	10 μm
Alanine	0.05
Arginine	0.3
Alanyl-glutamine	1.0
Asparagine	0.05
Aspartic acid	0.05
Cysteine	0.05
Glutamic acid	0.05
Glycine	0.05
Histidine	0.1
Isoleucine	0.2
Leucine	0.2
Lysine	0.2
Methionine	0.05
Phenylalanine	0.1
Proline	0.05
Serine	0.05
Taurine	0.05
Threonine	0.2
Tryptophan	0.02
Tyrosine	0.1
Valine	0.2
Phenol red	4.8 mg/L
Gentamicin	10 ug/mL
